# *Julbernardia paniculata* and *Pterocarpus angolensis*: From Ethnobotanical Surveys to Phytochemical Characterization and Bioactivities Evaluation

**DOI:** 10.3390/molecules25081828

**Published:** 2020-04-16

**Authors:** Eugénia Solange Santos, Ângelo Luís, Joana Gonçalves, Tiago Rosado, Luísa Pereira, Eugenia Gallardo, Ana Paula Duarte

**Affiliations:** 1 Centro de Investigação em Ciências da Saúde (CICS-UBI), Universidade da Beira Interior, Avenida Infante D. Henrique, 6200-506 Covilhã, Portugal; eugenia.santos@ubi.pt (E.S.S.); angelo.luis@ubi.pt (Â.L.); janitagoncalves@hotmail.com (J.G.); tiagorosadofful@hotmail.com (T.R.); egallardo@fcsaude.ubi.pt (E.G.); 2 Instituto Superior Politécnico da Huíla, Universidade Mandume Ya Ndemufayo, Bairro Comercial, Avenida Hoji Ya Henda N. 30, Caixa Postal N. 201, Lubango, Huíla, Angola; 3 Laboratório de Fármaco-Toxicologia, UBIMedical, Universidade da Beira Interior, Estrada Municipal 506, 6200-284 Covilhã, Portugal; 4 C4-Cloud Computing Competence Centre, UBIMedical, Universidade da Beira Interior, Estrada Municipal 506, 6200-284 Covilhã, Portugal; 5 Centro de Matemática e Aplicações (CMA-UBI), Universidade da Beira Interior, Rua Marquês d’Ávila e Bolama, 6201-001 Covilhã, Portugal; lpereira@ubi.pt

**Keywords:** *Julbernardia paniculata*, *Pterocarpus angolensis*, ethnobotanical surveys, HPLC-DAD, GC-MS, phytochemicals, wound-healing activity

## Abstract

*Julbernardia paniculata* and *Pterocarpus angolensis* are two plant species with important application in African traditional medicine, particularly in Angola, in the treatment of several diseases. However, scientific studies concerning these species are scarce. The goal of this work was to know better which medicinal approaches are used by the Huíla population in Angola by means of ethnobotanical surveys. Furthermore, extracts of both plants were phytochemically characterized. Antioxidant, anti-inflammatory, wound-healing activities, and potential cytotoxicity were also studied. With this study it was possible to verify that 67% of the individuals that use medicinal plants are women, and their main therapeutic uses are the treatment of problems of the digestive system and skin disorders. Barks of *J. paniculata* and leaves of *P. angolensis* are the most often used plant parts. Through high-performance liquid chromatography coupled to diode-array detector (HPLC-DAD) and GC-MS it was possible to characterize the chemical composition of the two species, which are rich in phenolic compounds, terpenes, terpenoids, sesquiterpenoids and fatty acids. Both plants showed to possess antioxidant, anti-inflammatory proprieties, and wound-healing activity. To the best of our knowledge, this is the most comprehensive study of these two species and the first ethnobotanical and ethnopharmacological study of medicinal plants from this region of Angola.

## 1. Introduction

Almost 80% of the world’s population depends on plant remedies for primary health care needs [1,2]. This is because modern hospital treatments are expensive compared to traditional and complementary medicines that are locally available and cheaper. Moreover, patients whose disease did not respond to conventional drugs or, also, due to cultural reasons often opt for traditional and complementary medicines [3]. The use of plants for their medicinal properties is a very old practice. It finds its origins in the oldest civilizations and has been preserved for many centuries across the world [4]. Popular knowledge of plants used by humans is based on the experience of thousands of years. By trial and error, people learnt how to recognize and use plants, including those with magic–religious functions [5]. The traditional medicine all over the world is nowadays revalued by extensive research activity on different plant species and their therapeutic principles [6].

The importance of African traditional medicine in the management of diseases has already been demonstrated [7]. Angolan folk medicine is an example of traditional medicine that is rich in plants with biological activities that are the result of centuries of trial and error experiments. Angolan folk medicine also displays a remarkable potential of bioactive compounds. Various studies revealed the activity of Angolan medicinal plants in human, animal, and plant pathogens [8].

*Julbernardia* (*Fabaceae*, *Caesalpiniaceae*) is a tropical African genus of miombo woodland with six species, of which *Julbernardia paniculata* (Benth.) Troupin is the most abundant in Angola, presenting several ethnobotanical applications [9,10]. The barks of this woody tree species are usually collected by local healers as a source of products having medicinal properties. Hence, the bark is often removed as a bulk product to be further processed for subsequent use, or as raw material from which flavorings or medicinal compounds are extracted to treat various ailments [11].

*Pterocarpus* genus is comprised of 100 species, amongst which is included, *Pterocarpus angolensis* (DC), (*Fabaceae*, *Papillionoideae*). *P. angolensis* is mainly found in the tropical regions of Africa as well as the Neotropical regions and Indomalaya [12,13], being the dominant member of the miombo woodland association and found scattered throughout the large area covered by this broad type of vegetation [13,14,15]. In folk medicine, *P. angolensis* medicinal barks are mainly used against gastrointestinal and urine-genital ailments, fertility problems, respiratory conditions, and skin disorders [12,16]. Extracts of *P. angolensis* are also commonly used by traditional healers in various regions of Africa for the treatment of malaria, gonorrhea, headaches, stomach aches, diarrhea, mouth sores and rashes [17]. This deciduous tree is treasured for its effectiveness against a wide range of diseases and is proving to be useful due to its high concentration of tannins and flavonoids [3,18]. Furthermore, *P. angolensis* was reported to exhibit anti-inflammatory activity and high inhibitory activity against *Staphylococcus aureus*, *Escherichia coli* and *Salmonella* Typhimurium [3,18]. It was also demonstrated that extracts of *P. angolensis* exhibited antiplasmodial activity, suppressing the chaperone activities and ATPase functions of the two parasite cytosol localized chaperones [19].

In this context, this work aimed the ethnobotanical study of *J. paniculata* and *P. angolensis* in the Huíla province of Angola together with the study of the extracts of both barks and leaves of these species. The extracts were characterized in what concerns flavonoids and total phenolic compounds. The phenolic composition was further determined by high-performance liquid chromatography coupled to diode-array detector (HPLC-DAD) and GC-MS. The antioxidant and anti-inflammatory activities of the extracts were then evaluated. Additionally, the wound-healing activity and potential cytotoxicity of selected extracts were also studied. To the best of our knowledge, this is the most comprehensive study of these two species and the first ethnobotanical and ethnopharmacological study of medicinal plants from this region of Angola.

## 2. Results and Discussion

### 2.1. Ethnobotanical Surveys

In the present ethnobotanical study, a total of 106 inhabitants of the Huíla province were interviewed. It was noticed that people between 30 and 40 years-old use plants the most (33%), while the age group under 20 years presented the lowest utilization rate (2%) (Figure S1A). These results suggest that young people are healthier and do not need remedies. In the province covered by this study, 67% of the individuals that use medicinal plants are women (Figure S1B), which could be explained by the tradition of being the women that prepare plant-based medicines cares for the family. This knowledge is believed to have passed down across generations, typically from mothers to daughters. Similar results were found previously on ethnobotanical studies in other regions of Africa [4]. It was also possible to verify that 63% of people using medicinal plants were non-native to the Huíla province (Figure S1C), which is probably due to the oral transmission of the information about the plants by their native users.

The ethnobotanical study revealed that there was no difference in what concerns the obtention forms of the medicinal plants. They can be either cultivated (36%), spontaneous (34%) or purchased (30%) (Figure S2A). Additionally, plants were collected both at rainy and dry seasons (64%) (Figure S2B), indicating that the use of medicinal plants does not depend on the weather and is continuous throughout the year.

The use of medicinal plants in the Huíla province was mainly due to experience of others (70%) (Figure S3A), which agrees with what is known about traditional and alternative medicines that are mostly transmitted orally within a specific community. Furthermore, the results indicated that people made use of books (7%) to learn about the medicinal plants (Figure S3A). Concerning the plant conservation mode, 59% of the inquired people used medicinal plants immediately after its collection (Figure S3B), possibly because it is easy to go out and collect them. There was also the practice of sun-drying (15%) and shade-drying (9%) the plants, which allows its medium- to long-term conservation.

The present survey proved that the most cited plants belongs to the *Fabaceae* family (20.28%) (Figure S4A), which is an economically important family of flowering plants. It includes trees, shrubs and perennial or annual herbaceous plants that are easily recognized by their fruit (legume) [20,21,22]. Considering the therapeutic indications of the medicinal plants, they were mostly employed to treat problems of the digestive system (37%), followed by skin disorders (30%) (Figure S4B). Moreover, cardiac and respiratory systems, post childbirth complications, Malaria, urinary and nervous systems were also mentioned on the surveys.

Regarding the preparation method of the medicinal plants, most users (52%) indicated that infusion was preferred, followed by decoction (20%) and maceration (17%) (Figure S5A). Typically, these methods are chosen since they are easy to prepare, using water as solvent, as it was similarly mentioned in previous ethnobotanical studies [1,4]. Concerning the parts of the medicinal plants more frequently used, leaves (57%) and barks (15%) were the favorite ones (Figure S5B). Other parts of the plants were also mentioned: Barks, roots, fruits, and mixtures between diverse parts (leaves and barks, leaves and roots and leaves and flowers).

To further details about the statistical quantitative analysis of the ethnobotanical results please consult the Supplementary Materials.

### 2.2. Phytochemical Characterization and Phenolic Profile

*J. paniculata* and *P. angolensis* demonstrated to be important plant species in the Huíla province, according to the ethnobotanical surveys. Considering that there are few studies about these plants, particularly about *J. paniculata*, in the present work it was decided to determine the total phenolic compounds and flavonoids contents in crude methanolic extracts and their fractions (*n*-hexane, diethyl ether and water) of both barks and leaves. Additionally, the phenolic profile of the samples was determined by HPLC-DAD and GC-MS.

Some phenolic compounds are ubiquitous in all plants as secondary metabolites [23]. In this work the total phenolic compounds were estimated using the Folin-Ciocalteu’s reagent (Table 1). Although recent reports described the limitations of this method to determine the absolute value of phenolic content, it is still widely used for total phenolic quantitative purposes in similar contexts considering relative values for comparison between different samples. Fractions 3 (water) were the samples that presented the highest concentrations of these compounds, followed by the crude methanolic extracts, Fractions 2 (diethyl ether) and Fractions 1 (*n*-hexane). Polyphenols are polar compounds easily extracted with polar solvents like methanol. Using the Charaux—Paris partition method, several fractions with increasing polarities were obtained, leading to the increase of the concentration of phenolic compounds in water fractions (Fractions 3). Considering that *n*-hexane and diethyl ether are less polar solvents, it was expected that Fractions 1 and 2 presented a lower content in polyphenols. In general, the barks of *J. paniculata* are the samples presenting higher concentrations in total phenolic compounds. Fraction 3 of *J. paniculata* barks presented the highest amount in these compounds. Considering all the results, it is possible to verify that *J. paniculata* leaves are the samples with the lowest concentration in phenolic compounds, indicating that they are predominantly present in the barks of this plant species. Contrariwise to what is verified in *P. angolensis*, whose leaves generally presented more polyphenols than barks. Previous works reported the presence of these compounds on *P. angolensis* extracts [12,17,18,19].

Flavonoids have been reported to present many bioactive properties, including anti-inflammatory, antimicrobial, antioxidant, cytotoxic and antitumor activities [24]. These compounds were determined in crude methanolic extracts and its fractions using the complexation with aluminum (Table 1). Overall, flavonoids are present in greater amounts in leaves of both plant species, agreeing with previous findings [25]. Fractions 1 (*n*-hexane) seem to be the samples with more flavonoids, which can be related with the extraction of some interferents by *n*-hexane, since it is a lipophilic solvent that is not able to extract hydrophilic compounds like flavonoids. Generally, *P. angolensis* is the plant species that presents more flavonoids in its composition.

To further characterize the crude extracts and its fractions, the phenolic profile was analyzed by HPLC-DAD. Initially, the HPLC-DAD method was fully validated according to the guiding principles of the Food and Drug Administration (FDA) [26]. Linearity was tested in the concentration range from 1 to 250 µg/mL for all the studied compounds (*n* = 5). A determination coefficient (R^2^) value of at least 0.99 and the calibrators’ accuracy within a ±15% interval from the nominal value (except at the lower limit of quantification ±20%) were adopted as acceptance criteria. The limit of quantification was 1 µg/mL for all compounds. Table 2 and Table 3 resume the analysis of the phenolic compounds for both plants. It was verified that gallic acid is not abundantly present in the crude extracts and fractions of *J. paniculata* and *P. angolensis*, as it was also previously reported for *P. angolensis* extracts [19]. Similar results were observed for caffeic acid and taxifolin [19]. It is difficult to compare the results now obtained for *J. paniculata*, since, to the best of our knowledge, this is the first study reporting its phenolic profile. Interestingly, Fraction 2 (diethyl ether) of *J. paniculata* leaves and crude methanolic extract of *P. angolensis* leaves were the samples in which the highest concentration of phenolic compounds were identified; for that reason, these samples were selected to study their GC-MS profile (Table 4 and Table 5).

The Fraction 2 (diethyl ether) of *J. paniculata* leaves presented in its composition, terpenes, terpenoids, sesquiterpenoids and steroids; saturated and unsaturated fatty acids were also found (Table 4). Megastigma-5,7-diene-3,4,9-triol and stigmasta-5,22-dien-3-ol were compounds identified in this sample that are potential free radical scavengers, possessing also anti-diabetic and anticancer properties [12]. Moreover, lup-20(29)-en-3β-ol was identified as 28.860% of the total. This compound is a pharmacologically active triterpenoid. It has several potential medicinal properties, namely antiprotozoal, antimicrobial, anti-inflammatory and anti-cancer activities [12]. β-Amyrin, a triterpene commonly detected in hemp fibers [27], was also detected. As far as we know, this is the first report about the GM-MS analysis of *J. paniculata* extracts. Regarding the crude methanolic extract of *P. angolensis* leaves (Table 5), the GC-MS results revealed the presence of hexadecanoic acid and lup-20(29)-en-3β-ol, as it was also found previously in *P. angolensis* extracts [12,18]. Hexadecanoic acid is known for its antibacterial and antifungal activities, while lup-20(29)-en-3β-ol presents several bioactivities, as was mentioned above [12]. 9-Octadecenamide, an amide fatty acid, is also present and is usually identified in spices [28].

Future research with these two plant species should focus on their chemical composition, which is directly related to their potential bioactivities and ethnobotanical uses.

### 2.3. Antioxidant Activity

The potential of the antioxidant compounds from plant origin for the preservation of health and protection from cancer and coronary heart disease is rising interest among researchers and food manufacturers as consumers move toward functional foods with specific health benefits [29]. In this sense, the antioxidant activity of the crude extracts and its fractions of both plant species was evaluated by two different methods (Table 6).

A 2,2-diphenyl-1-picrylhydrazyl (DPPH) free radical scavenging assay is routinely employed for the assessment of free radical scavenging potential of an antioxidant molecule and is considered as one of the standard and easy colorimetric methods to evaluate the antioxidant properties of plant extracts, essential oils and pure compounds [30].

Observing the results (Table 6), it is possible to conclude that barks of *J. paniculata* have the greatest antioxidant activity as measured by the DPPH method. Particularly, the crude methanolic extract of *J. paniculata* barks is the sample that presented the highest value of antioxidant activity index (AAI) (8.21), corresponding to a “very strong” antioxidant activity. These results could be related with the phenolic composition of this plant that presented a high amount of these compounds, as was mentioned above. Generally, the extracts and fractions of *P. angolensis* also presented the capacity to scavenge the free radicals.

β-carotene bleaching test is one of the most widespread methods applied for the evaluation of the antioxidant activity of plant extracts. In this test, the antioxidant activity is determined by comparing two competitive chemical reactions in which the examined antioxidant and/or model antioxidant, β-carotene, take part [31]. This method is also useful in the analysis of the capacity of the samples to inhibit the lipid peroxidation. The results obtained (Table 6) also show the greatest antioxidant activity of *J. paniculata* barks, since they presented lower values of IC_50_, except Fraction 1 (*n*-hexane). Moreover, the crude methanolic extract of *P. angolensis* barks also presented similar antioxidant activity.

Taken together, the results now obtained for the antioxidant activity and phenolic composition of *J. paniculata* support further research with this plant.

### 2.4. Anti-Inflammatory Activity and Cytotoxicity

Inflammation is the physiological process that initiates in response to bacterial infection or tissue damage [32]. Denaturation of proteins is one of the causes of inflammation, leading to production of autoantigens. The mechanism of denaturation possibly involves changes in electrostatic, hydrogen, hydrophobic and disulfide bonding [33].

In the present work, the anti-inflammatory activity of the samples was evaluated by the capability of inhibiting protein denaturation. The selection of the samples was based on the results of ethnobotanic surveys together with the results of the phytochemical characterization. Since the barks of *J. paniculata* and the leaves of *P. angolensis* were shown to be more frequently used by people from the Huíla province, these samples were chosen to additionally evaluate its anti-inflammatory activity, cytotoxicity, and wound-healing activity. Considering the phytochemical characterization, the crude methanolic extracts and Fractions 3 (water) of those samples were selected, because they presented higher contents in bioactive compounds.

The results of anti-inflammatory activity (Table 7) showed that *P. angolensis* samples did not present that activity, contrariwise to what was verified for *J. paniculata* samples, whose Fraction 3 presented the best anti-inflammatory activity, which is probably due to the high concentration of total phenolic compounds found in that sample.

Concerning the results of cytotoxicity (Table 7), determined as the percentage of cell viability in the presence of different concentration of the samples, it was verified that crude methanolic extract (250 and 750 mg/L) and Fraction 3 (water) (500 mg/L) of *J. paniculata,* and all the tested samples of *P. angolensis* presented a significant effect (*p*-value < 0.05) in cell viability (Table 7). These results suggest that extracts of *J. paniculata* and *P. angolensis* may have a potential in vitro cytotoxicity at the tested concentrations.

### 2.5. Wound-Healing Activity

Wounds were characterized, based on the nature of repair process, as acute and chronic. Completely healed wounds within twelve weeks with minimal scarring were referred to as acute [34]. Chronic wounds, such as venous, arterial, pressure and diabetic ulcers, were often associated with advanced age, patient immobility, compromised blood circulation and systemic illnesses [35].

Wound-healing is a process of reconstruction of injured skin, coordinated by interaction of various epithelial and mesenchymal cells with cytokines, chemokines and growth factors [36].

Natural products play a major role in proliferation of fibroblasts and keratinocytes. Plant extracts were reported to contain growth factors, cell signaling molecules and cell adhesion molecules [36].

**Figure 1:**
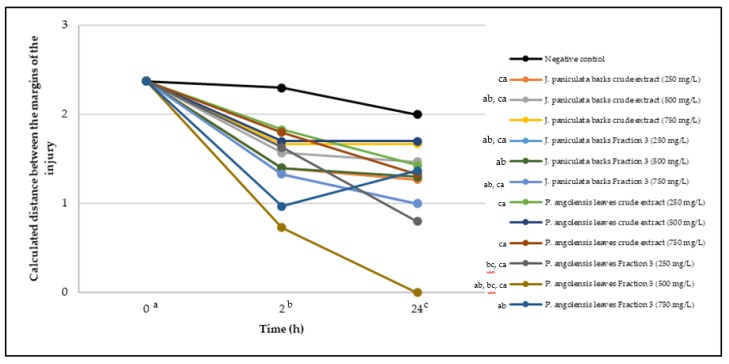
Calculated distance between the margins of the injury (upper letters were used to identify the statistical comparisons, indicating in the samples the pairs that presented significant results (*p*-value < 0.05)).

In the present work, the wound-healing potential of *J. paniculata* and *P. angolensis* was evaluated by the wound scratch assay. Regarding the evolution of the calculated distance between the margins of the injury in the presence of the selected samples at different concentrations (Figure 1), significant differences were observed:

- Between 0 and 2 h after the incubation with the samples: *J. paniculata* crude methanolic extract of barks (500 mg/L) and the respective Fraction 3 (250, 500 and 750 mg/L) and *P. angolensis* Fraction 3 of leaves (500 and 750 mg/L);
- Between 0 and 24 h after the incubation with the samples: *J. paniculata* crude methanolic extract of barks (500 mg/L) and the respective Fraction 3 (250 and 750 mg/L), *P. angolensis* crude methanolic extract of leaves (250 and 750 mg/L) and the respective Fraction 3 (500 mg/L);
- Between 2 and 24 h after the incubation with the samples: *P. angolensis* Fraction 3 of leaves (250 and 500 mg/L).

These results clearly show that the size of the injuries decreases in the presence of the samples, which indicates the potential wound-healing activity of *J. paniculata* and *P. angolensis.* Furthermore, the average distance of the negative control wounds was significantly higher than the average distance of the wounds where the samples were applied (Table 8), corroborating the above-mentioned wound-healing activity of these two plant species.

Table 9 shows the fibroblasts migration in the presence of the *J. paniculata* samples with different concentrations. Photographs indicating comparative cell migration in the presence of the *P. angolensis* samples with different concentrations are summarized in Table 10.

Observing all the images, it is possible to verify that the injuries close slowly with time in the presence of the samples, which is in agreement with the calculated distance between the margins of the injury previously calculated. Either single or multiple mechanisms could be responsible in different phases of wound-healing which can contribute to the overall outcome of the wound-healing process [37].

The conventional assays to determine the efficacy of plant extracts for wound-healing comprises painful invasive procedures in animal models. The in vitro assay used in this study allowed the screening of various samples having antioxidant and anti-inflammatory activities essential for wound-healing. It was reported earlier that wound-healing and antioxidant properties co-exist in plant extracts [36].

## 3. Materials and Methods

### 3.1. Ethnobotanical Surveys

#### 3.1.1. Huíla Province Geo-Ethnographical Profile

**Figure 2:**
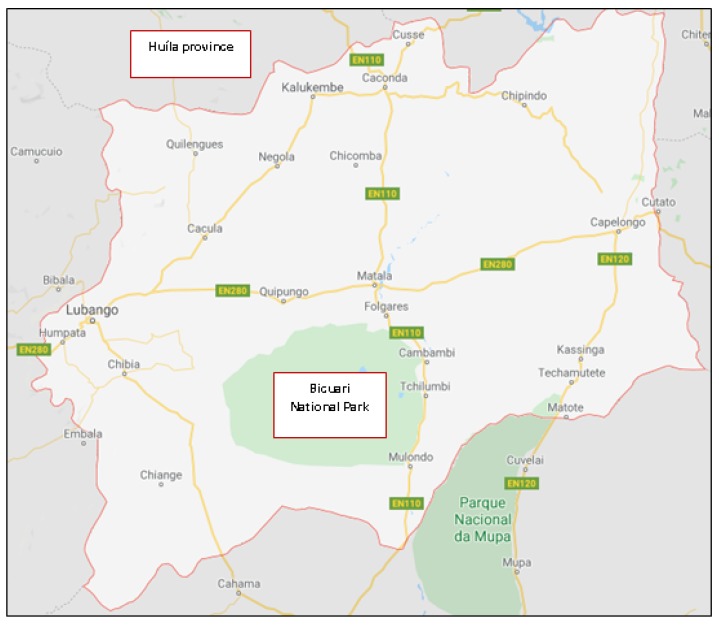
Study area showing Huíla province and Bicuari National Park (adapted from Google Maps, https://www.google.pt/maps/preview).

The ethnobotanical surveys took place in the Huíla province of Angola (Figure 2). Huíla is one of the 18 Angolan provinces and it has an area of 79,023 km^2^ and a population of 3,334,456 inhabitants, being Lubango its capital. Basket-making is an important activity in the province; many people make baskets out of reeds. Huíla is crossed by the northwesterly line of equal longitude and latitude. This province is bordered on the west by the provinces of Namibe and Benguela, to the north by Bié and Cuando Cubango, and to the south by the province of Cunene. The winding road known as Leba Hill, as well as Bicuari National Park (Figure 2) are in the Huíla province. Bicuari National Park was established in 1964 and covers an area of 790 km^2^. According to the Köppen—Geiger climatic classification, the oceanic climate predominates in the province. The climate is, in general, hot, with uniform rains during the year and an average annual temperature above 20 °C. In the areas located at a higher altitude, the climate can be classified as maritime temperate. The main language spoken in this province is Portuguese, with the southern dialect variant being registered, one of the four within Angolan Portuguese. Among the traditional ones, the largest presence is of the Nhaneca language. Ethnologically, the population of the Huíla province is diverse, since it consists mainly of four ethnolinguistic groups, namely: Nyaneca-Nkhumbi, Ovimbundu, Ngangela and Herero [38].

#### 3.1.2. Field Interviews

The surveys were conducted over a period of six months (second semester of 2017), through a semi-structured questionnaire. A total of 106 native people was face-to-face interviewed at their homes without limit of time. The questionnaire was mainly focused on the ethnobotanical allegations and traditional believes of local communities and nearby people, but also includes the profile of the respondents like age and gender.

#### 3.1.3. Collection and Identification of Plant Materials

Samples of *J. paniculata* and *P. angolensis* were collected in Bicuari National Park (Figure 2) (GPS coordinates: 15°22′47.4″ S 14°45′10.3″ E; Altitude: 1250 m) during January and February of 2018. The plant specimens were identified by a botanist from Instituto Superior de Ciências da Educação da Huíla, Universidade Mandume Ya Ndemufayo, Huíla, República de Angola. The identified plants were prepared to be deposited in herbarium and stored in a specific laboratory of Instituto Superior Politécnico da Huíla, Universidade Mandume Ya Ndemufayo, Huíla, República de Angola.

#### 3.1.4. Quantitative Analysis of the Ethnobotanical Results

In order to analyze the collected data and to interpret the respective results, a data base was firstly prepared using Microsoft Excel (Windows, Washington, DC, USA). Then, the subsequent quantitative value indexes were calculated using the statistical program SPSS version 25 (IBM, Portsmouth, UK).

##### Informant Consensus Factor

The informant consensus factor (ICF) was obtained using the Equation (1):

ICF = (N_ur_ − N_t_)/(N_ur_ − 1) (1)

where N_ur_ refers to the total number of use reports for each category of diseases, and N_t_ is the number of taxa used in that category. It is used to test the homogeneity of knowledge on the use of species in the illness categories between the population. The ICF provides a range from 0 to 1. High ICF shows that there is a narrow well-defined group of species used to treat a particular ailment category and/or that information is exchanged between informants. Low ICF values (close to zero) indicate that informants disagree over which plant to use due to random choosing or lack of exchange of information about the use among informants [1,4].

##### Fidelity Level

The fidelity level (FL) index was calculated using the Equation (2) to determine the most preferred species used in the treatment of a particular ailment, since more than one plant species are used in the treatment in the same category:

FL = (N_p_/N × 100) (2)

where N_p_ is the number of informants citing the use of the plant for a particular illness, and N is the total number of informants citing the species for any illness. High FL indicates high frequency of use of the plant species for treating a particular ailment category by the informants of the study area [1,4].

##### Relative Frequency Citation

The relative frequency citation (RFC) index was determined by using the Equation (3):

RFC = FC/N(3)

This index is obtained by dividing the number of informants mentioning a useful species—frequency of citation (FC)—by the total number of informants in the survey (N). RFC varies from 0 (when nobody refers to a plant as a useful one) to 1 (when all the informants mention it as useful). RFC index, which does not consider the use-category or use-report is a single record for use of a plant mentioned by an individual [1,4].

##### Use Value

The use value (UV) demonstrates the relative importance of plants known locally. It was calculated using the Equation (4):

UV = Σ U_i_/N (4)

where U_i_ is the number of uses mentioned by each informant for a particular species, and N is the total number of informants. The UV is applied in determining the plants with the highest use (most frequently indicated) in the treatment of an ailment [1,4].

### 3.2. Extraction and Fractionation

After the collection of the plants, they remained at room temperature until fully dry. Then, both barks and leaves of *J. paniculata* and *P. angolensis* were reduced to coarse powder (<2 mm) using a laboratory cutting mill. The samples (10 g) were extracted with 200 mL of methanol at 35 °C in an ultrasound-bath during 1 h with frequent shaking. After filtration, the residues were re-extracted under the same conditions for more three times. Subsequently, the extracts were centrifuged (7000× *g*, 20 min) and then concentrated using a rotary evaporation system (40 °C) [39,40].

**Figure 3:**
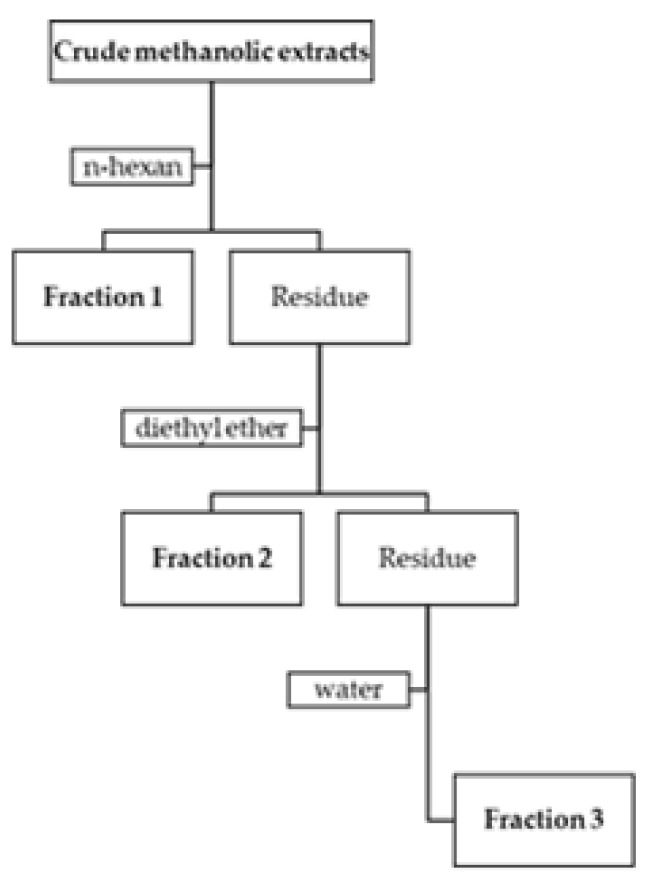
Schematic diagram showing the sequential liquid-liquid partition of the crude methanolic extracts.

The crude methanolic extracts were fractionated using the Charaux–Paris method, which consists in a sequential liquid–liquid partition with *n*-hexane (Fraction 1), diethyl ether (Fraction 2) and water (Fraction 3) (Figure 3) [41,42]. This process allows obtaining fractions with different and increasing polarities.

### 3.3. Phytochemical Characterization and Phenolic Profile

#### 3.3.1. Total Phenolic Compounds Determination

Despite recent evidences suggesting that the substrate specificity of Folin-Ciocalteu’s reagent is much broader, including vitamin C, some amino acids, the total phenolic compounds were determined by Folin-Ciocalteu’s colorimetric method [25,43], using gallic acid as standard. Initially, 450 μL of distilled water were mixed with 50 μL of crude extracts and fractions diluted in methanol or gallic acid solutions. Then, 2.5 mL of Folin-Ciocalteu’s reagent (0.2 N) were added, being the mixtures left for 5 min before the addition of 2 mL of aqueous Na_2_CO_3_ (75 g/L). The reaction mixtures were incubated for 90 min at 30 °C. After incubation, the content in total phenolic compounds was determined by colorimetry at 765 nm. A standard curve was prepared using methanolic solutions of gallic acid (y = 0.0010x; R^2^ = 0.9612). The total phenolic compounds content was expressed as mg of gallic acid equivalents (GAE)/g of sample (extracts or fractions) [25,43].

#### 3.3.2. Flavonoids Determination

The aluminum chloride colorimetric method was used to determine the flavonoids content according to a previously implemented method [25,43]. To 500 μL of each solution, either crude extracts and fractions diluted with methanol or quercetin (used as standard), 1.5 mL of methanol, 0.1 mL of aluminum chloride 10% (*w*/*v*), 0.1 mL of 1 M potassium acetate and 2.8 mL of distilled water were added. These solutions remained for 30 min at room temperature and then the absorbances were measured using a spectrophotometer at 415 nm. To construct the calibration curve, quercetin solutions were prepared in methanol (y = 0.0146x; R^2^ = 0.9887). The flavonoids content was expressed as mg of quercetin equivalents (QE)/g of sample (extracts or fractions) [25,43].

#### 3.3.3. HPLC-DAD Analysis

In order to identify and quantify the phenolic compounds more representative present in the crude extracts and fractions, a simple methodology for the simultaneous determination of the compounds by HPLC-DAD was developed and validated. Working solutions of each phenolic compound (gallic acid, chlorogenic acid, caffeic acid, vanillic acid, syringic acid, *p*-coumaric acid, taxifolin, rutin, ferulic acid, ellagic acid, rosmarinic acid and quercetin) were prepared by dilution with methanol to the final concentration of 1 mg/mL. These solutions were stored in the absence of light at 4 °C until further use.

A high-performance liquid chromatography system (HPLC) with a binary pump coupled to a diode-array detector (DAD) from Agilent Technologies (Soquimica, Lisboa, Portugal,) was used. The compounds were separated with an YMC-Triart PFP (5 µm, 4.6 by 150 mm) analytical column coupled to a Guard-c holder (4 by 10 mm) containing Triart PFP (5 µm, 3 by 10 mm) pre-column, all from YMC Europe GMBH.

A gradient mobile phase system consisting of (A) acetonitrile and (B) 0.1% trifluoroacetic acid was used. The separation was achieved with a linear gradient program as follows: 10% *v*/*v* (A) from 0 to 3 min; ramp up to 15% *v*/*v* (A) from 3 to 15 min; holds at 15% *v*/*v* (A) until 20 min; ramp up to 18% *v*/*v* (A) from 20 to 25 min; then up to 30% *v*/*v* (A) from 25 to 40 min; up to 50% *v*/*v* (A) from 40 to 45 min; and up to 100% *v*/*v* (A) from 45 to 50 min; after which it returned to the initial conditions, 10% *v*/*v* (A), from 50 to 55 min. The flow rate was 1.0 mL/min. The column and sampler temperatures were set at 35 and 4 °C, respectively. The injection volume was 50 µL and the analytes were detected between 280 and 360 nm. Table 11 summarizes the retention times and wavelengths for each phenolic compound. The dried samples (20 mg) were dissolved in 1 mL of absolute ethanol, centrifuged (20,000× *g*) and then filtered (0.22 µm) before being transferred to the autosampler for injection into the HPLC-DAD system. The concentration of the compounds was expressed as µg/mg of sample (extracts or fractions), corresponding to two chromatographic profilings.

#### 3.3.4. GC-MS Analysis

Analysis was carried out on a gas chromatograph (GC) model HP7890B coupled to a mass spectrometer (MS) model 5977A from Agilent Technologies. The separation of the analytes was achieved using a 5% phenylmethylsiloxane (HP-5MS) capillary column (0.25 μm, 30 m by 0.25 mm) supplied by Agilent Technologies. The carrier gas, helium, was set at a constant flow rate of 0.8 mL/min. The temperatures of the injector and detector were set at 240 °C and 280 °C, respectively. The ion source temperature was set at 230 °C and the quadrupole’s at 150 °C. The oven temperature started at 150 °C for 1 min increasing 5 °C/min up to 290 °C hold for 8 min. The mass spectrometer was operated in electron ionization mode with an energy of 70 eV and emission current of 300 μA. The injection volume was 2 μL in split mode (split ratio of 1:10). The identification of the analytes was performed in full-scan mode (*m*/*z* 50–550) using the GC-MS ChemStation Software (2011) from Agilent Technologies.

The selected samples (1 mg) were reconstituted in 1 mL of methanol, filtered on a 0.22 μm cellulose acetate pore filter, being 2 μL injected into the GC-MS system. The identification of the compounds by GC-MS was performed by comparing a query mass spectrum with reference mass spectra using mass spectral libraries (NIST17, PMW–TOX2 and the Wiley’s Flavors and Fragrances of Natural and Synthetic Compounds). Furthermore, the identified compounds (name and CAS number) were confirmed using the PubChem database (https://pubchem.ncbi.nlm.nih.gov/).

### 3.4. Biological Activities Evaluation

#### 3.4.1. Antioxidant Activity

##### DPPH Free Radical Scavenging Assay

The DPPH free radical scavenging assay was used to evaluate the antioxidant activity of the crude extracts and fractions [25]. Briefly, 100 µL of methanolic solutions of the samples at different concentrations were added to 3.9 mL of three DPPH methanolic solutions (0.2, 0.1242 and 0.08 mM). The control consisted in 100 µL of methanol mixed with 3.9 mL of each DPPH solution. After a 90 min incubation period at room temperature in the dark, the absorbances were measured at 517 nm. The radical scavenging activity was calculated as follows: %I = [(Abs_control_ − Abs_sample_)/Abs_control_] × 100, where Abs_control_ is the absorbance of the control and Abs_sample_ is the absorbance in the presence of the samples at different concentrations. The IC_50_ was calculated graphically using a curve in the linear range, by plotting the concentrations of the samples vs. the corresponding scavenging effect. The antioxidant activity was expressed as the antioxidant activity index, calculated as follows: AAI = (final concentration of DPPH in the control)/(IC_50_). The AAI allowed the classification of the antioxidant activity of the samples as: Poor (AAI ≤ 0.5), moderate (0.5 < AAI ≤ 1.0), strong (1.0 < AAI < 2.0) or very strong (AAI ≥ 2.0) [25,44].

##### β-Carotene Bleaching Test

After the preparation of a β-carotene solution (20 mg/mL in chloroform), 500 μL were added to linoleic acid (40 μL), Tween 40 (400 mg) and chloroform (1 mL). This mixture was then evaporated at 45 °C for 5 min in a rotary evaporation system to remove the chloroform and immediately diluted with distilled water saturated with O_2_ (100 mL). The water was added slowly to the mixture and this was vigorously shaken to form an emulsion. Then, this emulsion (5 mL) was transferred into test tubes containing the crude extracts or fractions in methanol at different concentrations (300 μL). The control consisted in 5 mL of the emulsion and 300 μL of methanol. The tubes were then gently shaken and placed at 50 °C in a water bath for 1 h. The absorbances were measured at 470 nm, using a spectrophotometer (Helios—Omega, Thermo Scientific, Waltham, MA, USA), against a blank consisting of an emulsion without β-carotene. The measurements were carried out at initial time (t = 0 h) and at final time (t = 1 h). The antioxidant activity was measured in terms of percentage of inhibition of β-carotene’s oxidation by: %I = (Abs^t = 1 h^_sample_ − Abs^t = 1 h^_control_)/(Abs^t = 0 h^_control_ − Abs^t = 1 h^_control_), where Abs^t = 1 h^ is the absorbance of the sample or control at final time of incubation and Abs^t = 0 h^ is the absorbance of the control at initial time of incubation [25].

#### 3.4.2. Anti-Inflammatory Activity

The anti-inflammatory activity was determined by evaluating the capacity of the selected samples to inhibit protein denaturation [45]. Initially, a solution of bovine serum albumin (BSA) at 1% (w/v) in phosphate buffer saline (PBS) was prepared. The pH of this solution was adjusted to 6.8 using glacial acetic acid. Then, 100 µL of the samples diluted in dimethyl sulfoxide (DMSO) were mixed, in test tubes pre-heated at 37 °C, with 900 µL of the BSA solution previously prepared. The control was composed of distilled water. The tubes were then incubated for 10 min at 72 °C and after this period cooled in ice for another 10 min. Finally, measurements of the absorbances were performed using a microplate reader (BIO-RAD, Hercules, CA, USA) at 620 nm. The percentage of inhibition of protein denaturation was calculated using the following equation: %I = 100 − [(Abs_sample_ × 100)/Abs_control_), where Abs_control_ is the absorbance of the control and Abs_sample_ is the absorbance of each sample [45].

#### 3.4.3. Cytotoxicity

Normal human dermal fibroblasts (NHDF) cell line was maintained in RPMI-1640 culture medium supplemented with 10% fetal bovine serum (FBS), 1% mixture of antibiotic/antimycotic, 0.01 M of HEPES, 0.02 M of *L*-glutamine and 0.001 M of sodium pyruvate. Subsequently, the cells were incubated at 37 °C in an air incubator with a humidified atmosphere with 5% CO_2_ [46].

The cytotoxicity of the selected samples was determined by the cell viability assay using the MTT method after 24 h of incubation with the samples. This method is based on the reduction of 3-(4,5-dimethylthiazol-2-yl)-2,5-diphenyltetrazolium bromide (MTT) into formazan crystals. The cells were seeded in 96-well plates (5 × 10^3^ cells/well), which after reaching confluence were exposed to the samples dissolved in RPMI-1640 culture medium. Supplemented RPMI-1640 culture medium was added to the negative control wells. At the end of incubation, the medium in the wells was removed and replaced by the MTT solution and incubated again at 37 °C for 4 h. Afterwards, medium-containing MTT was removed and formazan crystals were dissolved in DMSO, being the absorbances recorded using a microplate reader at 570 nm [46].

#### 3.4.4. Wound-Healing Activity

The selected samples were tested for wound-healing activity by using the wound scratch assay [35,47]. NHDF cells were seeded in 12-well plates (4 × 10^4^ cells/well) and cultured until a monolayer confluence was reached. After the adhesion of the cells, the medium was removed from the wells and the cell monolayer was scraped in a straight central line using a p200 micropipette tip, creating a scratch, being reference points marked in the plates. The wells were washed with PBS to remove floating cells and cell debris. Then, the PBS was removed, and the samples prepared in RPMI-1640 and sonicated were added to the wells. Supplemented RPMI-1640 culture medium was added to the negative control wells. After this, the plates were placed under a phase-contrast microscope and images were acquired at the initial moment (t = 0 h). Then, the plates were incubated at 37 °C (5% CO_2_) and checked once again under the microscope after 2 and 24 h [35,47]. The size of the scratch zones was assessed manually using a digital image analysis tool (IC Measure software version 2.0.0.161, The Imaging Source, Bremen, Germany) that allowed the estimation of the distance between the injury margins.

### 3.5. Statistical Analysis

In general, the results were presented as mean values ± standard deviation (SD). To determine the reproducibility of the measurements, each assay was performed at least in triplicate. The data were analyzed using the statistical program SPSS version 25 and a *p*-value < 0.05 was considered significant. One-way analysis of variance (ANOVA) was undertaken to test for significant differences among means for total phenolic compounds, flavonoids, antioxidant, and anti-inflammatory activities. The results of cell viability were expressed as median and range and were tested using the non-parametric test of Wilcoxon—Mann—Whitney. The results of the wound-healing activity were analyzed by two tests: (1) repeated measures ANOVA was employed to analyze the efficacy of the samples in the evolution of the distance of the injuries in the three moments (0, 2 and 24 h); (2) multiple comparison of means with Bonferroni correction was used to identify which pairs of means differ from each other, for the interaction between groups and moments.

## 4. Conclusions

The ethnobotanical study presented in this work shows that the most dominant plant family used in traditional medicine in Huila province of Angola is *Fabaceae* (20.28%), the parts of the plants most frequently used are leaves (57%) and barks (15%), and most users (52%) indicated that infusion is the preferred, followed by decoction (20%).

This work presented an overview of the phytochemical composition and bioactivities of *J. paniculata* and *P. angolensis*, the two most used plant species, as the results of ethnobotanical surveys demonstrated. These plants showed a potential wound-healing activity, thus confirming their traditional use in skin disorders, namely the use in repairing skin lesions and regeneration of damaged tissues. In addition, their antioxidant and anti-inflammatory properties can contribute to this beneficial effect. These biological activities are also probably the basis for the use of these plants in digestive systems ailments, with this use being mentioned in 37% of the surveys.

As a result of the present study, *J. paniculata* and *P. angolensis* demonstrated important biological properties, antioxidant, anti-inflammatory and wound-healing activities, which seems to be related with their phenolic profile, but it is recommended to perform further phytochemical characterization, as well as further investigation concerning digestive system mechanism (both intestinal bacteria and enzymes) and the potential cytotoxicity.

## Figures and Tables

**Table 1:** Total phenolic compounds and flavonoids contents.

Plant Species	Plant Part	Samples	Phenolic Compounds (mg GAE/g Sample) ^1^	Flavonoids (mg QE/g Sample) ^1^
*J. paniculata*	Barks	Crude extract	410.93 ± 16.72 g	1.64 ± 0.19 a
		Fraction 1	19.88 ± 2.72 a	57.69 ± 3.25 f
		Fraction 2	366.13 ± 7.94 f	3.07 ± 0.18 a
		Fraction 3	703.73 ± 13.29 h	2.76 ± 0.02 a
	Leaves	Crude extract	77.60 ± 6.80 b	18.65 ± 0.79 c
		Fraction 1	18.89 ± 0.84 a	62.21 ± 0.82 g
		Fraction 2	39.20 ± 2.26 a	61.89 ± 2.81 f,g
		Fraction 3	188.67 ± 4.41 c	12.68 ± 0.18 b
*P. angolensis*	Barks	Crude extract	258.40 ± 5.81 d	2.25 ± 0.20 a
		Fraction 1	6.83 ± 1.00 a	71.97 ± 2.17 h
		Fraction 2	173.07 ± 13.48 c	10.79 ± 0.31 b
		Fraction 3	305.73 ± 13.71 e	4.47 ± 0.21 a
	Leaves	Crude extract	262.00 ± 1.70 d	28.48 ± 1.26 d
		Fraction 1	93.33 ± 6.07 b	68.10 ± 3.05 h
		Fraction 2	257.00 ± 18.95 d	45.78 ± 3.18 e
		Fraction 3	360.93 ± 12.17 f	23.41 ± 0.90 c,d

^1^ Mean values in a column with different letters (a to h) are significantly different (*p*-value < 0.05).

**Table 2:** Phenolic profile of *J. paniculata* (*n* = 2; N.D. = not detected).

Phenolic Compounds (µg/mg Sample)	Barks				Leaves			
	Crude Extract	Fraction 1	Fraction 2	Fraction 3	Crude Extract	Fraction 1	Fraction 2	Fraction 3
Gallic acid	N.D.	N.D.	N.D.	N.D.	0.13 ± 0.04	0.11 ± 0.03	N.D.	0.16 ± 0.01
Chlorogenic acid	0.21 ± 0.05	N.D.	0.20 ± 0.06	0.20 ± 0.05	N.D.	N.D.	N.D.	N.D.
Caffeic acid	N.D.	N.D.	N.D.	N.D.	N.D.	N.D.	0.16 ± 0.01	N.D.
Vanillic acid	N.D.	N.D.	N.D.	N.D.	N.D.	N.D.	0.96 ± 0.04	N.D.
Syringic acid	0.14 ± 0.04	N.D.	N.D.	N.D.	0.12 ± 0.01	0.10 ± 0.01	0.15 ± 0.02	N.D.
*p*-Coumaric acid	0.16 ± 0.01	N.D.	0.20 ± 0.02	0.15 ± 0.01	N.D.	N.D.	0.54 ± 0.03	0.18 ± 0.02
Taxifolin	0.07 ± 0.01	N.D.	0.16 ± 0.04	0.11 ± 0.01	0.12 ± 0.02	N.D.	N.D.	N.D.
Rutin	N.D.	N.D.	0.11 ± 0.02	N.D.	N.D.	N.D.	0.90 ± 0.15	0.39 ± 0.03
Ferulic acid	0.28 ± 0.02	N.D.	0.28 ± 0.02	N.D.	0.32 ± 0.03	0.28 ± 0.01	0.87 ± 0.02	0.38 ± 0.02
Ellagic acid	N.D.	N.D.	0.09 ± 0.01	N.D.	0.15 ± 0.03	0.06 ± 0.01	0.07 ± 0.01	0.14 ± 0.02
Rosmarinic acid	0.08 ± 0.01	N.D.	0.12 ± 0.03	0.08 ± 0.01	N.D.	0.07 ± 0.01	0.12 ± 0.02	0.07 ± 0.01
Quercetin	0.25 ± 0.03	N.D.	0.20 ± 0.01	0.19 ± 0.02	N.D.	0.18 ± 0.02	N.D.	N.D.
Total	1.18 ± 0.04	0.00 ± 0.00	1.36 ± 0.01	0.73 ± 0.09	0.84 ± 0.01	0.81 ± 0.08	3.78 ± 0.22	1.33 ± 0.09

**Table 3:** Phenolic profile of *P. angolensis* (*n* = 2; N.D. = not detected).

Phenolic Compounds (µg/mg Sample)	Barks				Leaves			
	Crude Extract	Fraction 1	Fraction 2	Fraction 3	Crude Extract	Fraction 1	Fraction 2	Fraction 3
Gallic acid	0.13 ± 0.01	N.D.	0.15 ± 0.03	0.12 ± 0.01	N.D.	N.D.	N.D.	N.D.
Chlorogenic acid	N.D.	N.D.	N.D.	N.D.	N.D.	N.D.	N.D.	N.D.
Caffeic acid	0.13 ± 0.02	N.D.	0.21 ± 0.01	0.13 ± 0.02	0.15 ± 0.01	N.D.	N.D.	N.D.
Vanillic acid	N.D.	N.D.	N.D.	N.D.	0.07 ± 0.01	N.D.	0.91 ± 0.07	N.D.
Syringic acid	0.11 ± 0.03	N.D.	0.14 ± 0.03	N.D.	N.D.	N.D.	N.D.	N.D.
*p*-Coumaric acid	N.D.	N.D.	0.17 ± 0.03	N.D.	0.34 ± 0.04	N.D.	0.39 ± 0.03	0.28 ± 0.04
Taxifolin	N.D.	N.D.	N.D.	N.D.	N.D.	N.D.	N.D.	N.D.
Rutin	N.D.	N.D.	N.D.	N.D.	39.87 ± 4.43	12.07 ± 1.44	14.50 ± 0.28	17.83 ± 2.64
Ferulic acid	0.27 ± 0.04	0.28 ± 0.05	0.34 ± 0.05	N.D.	N.D.	N.D.	0.57 ± 0.03	N.D.
Ellagic acid	N.D.	N.D.	N.D.	N.D.	N.D.	0.11 ± 0.01	0.28 ± 0.02	N.D.
Rosmarinic acid	N.D.	N.D.	0.18 ± 0.01	0.06 ± 0.01	0.43 ± 0.01	N.D.	0.71 ± 0.06	N.D.
Quercetin	N.D.	N.D.	N.D.	N.D.	N.D.	N.D.	N.D.	N.D.
Total	0.65 ± 0.01	0.28 ± 0.05	1.18 ± 0.03	0.31 ± 0.05	40.87 ± 4.47	12.18 ± 1.42	17.35 ± 0.12	18.12 ± 2.60

**Table 4:** Phytocomponents identified in the Fraction 2 (diethyl ether) of *J. paniculata* leaves by GC-MS.

Retention Time (min)	Compounds	Peak Area (% of Total)
3.416	Linalool oxide	0.331
4.276	2,6-Di(*t*-butyl)-4-hydroxy-4-methyl-2,5-cyclohexadiene-1-one	2.638
4.792	Phenol	3.076
5.223	2(4H)-Benzofuranone	0.348
6.458	1-Hexadecene	0.637
8.129	1-(P-Methoxyphenyl)-2-methoxyprop-1-ene	0.729
8.597	Loliolide	1.849
8.890	Atropine	0.651
8.939	Megastigma-5,7-diene-3,4,9-triol	0.580
9.594	Neophytadiene	6.087
10.726	1,4-Dihydrophenanthrene	0.506
11.085	Hexadecanoic acid	0.479
11.793	1,2-Benzenedicarboxylic acid	0.317
13.224	4-Oxazolecarboxylic acid	0.135
13.447	Menthol	0.545
14.164	Linolenic acid methyl ester	0.253
14.367	Phytol	1.996
15.501	Dodecanamide	0.383
18.427	Linoleic acid	0.485
18.521	9-Octadecenamide	3.376
21.848	Medicarpin	0.690
22.021	6H-Benzofuro [3,2-c][1]benzopyran-6a(11aH)-ol	0.377
26.436	1-Docosene	0.407
31.523	Stigmasta-5,22-dien-3-ol	1.003
32.557	23S-Ethylcholest-5-en-3-β-ol	6.932
32.721	3-Keto-urs-12-ene	3.758
32.830	Alnulin	1.112
33.171	β-Amyrin	5.714
33.646	D:C-Friedoolean-8-en-3-one	17.602
34.114	Lup-20(29)-en-3β-ol	28.860

**Table 5:** Phytocomponents identified in the crude methanolic extract of *P. angolensis* leaves by GC-MS.

Retention Time (min)	Compounds	Peak Area (% of Total)
9.590	Neophytadiene	0.584
11.087	7,9-Di-tert-butyl-1-oxaspiro [4.5]deca-6,9-diene-2,8-dione	0.275
12.092	Eicosamethylcyclodecasiloxane	0.901
15.483	Hexadecanamide	0.257
17.246	Eseroline	1.191
18.518	9-Octadecenamide	3.303
20.845	Hexadecanoic acid	1.409
32.534	Pregn-5-en-3-ol	1.578
34.079	Lup-20(29)-en-3β-ol	10.332

**Table 6:** Antioxidant properties of the samples.

Plant Species	Plant Part	Samples	DPPH Free Radical Scavenging Assay			β-Carotene Bleaching Test
			IC_50_ (mg/L) ^1^	AAI ^1^	Antioxidant Activity	IC_50_ (mg/L) ^1^
*J. paniculata*	Barks	Crude extract	5.51 ± 0.93 a	8.21 ± 0.19 e	Very strong	422.67 ± 18.53 a
		Fraction 1	39.06 ± 7.91 a	1.15 ± 0.19 a	Strong	1350.79 ± 287.89 a,b,c
		Fraction 2	6.60 ± 1.56 a	6.52 ± 0.52 d	Very strong	493.19 ± 34.15 a,b
		Fraction 3	7.35 ± 1.20 a	6.86 ± 0.44 d	Very strong	533.83 ± 1.55 a,b
	Leaves	Crude extract	57.62 ± 7.41 ab	0.75 ± 0.05 a	Moderate	1231.00 ± 269.36 a,b,c
		Fraction 1	184.54 ± 36.43 c	0.28 ± 0.05 a	Poor	8222.58 ± 186.22 f
		Fraction 2	68.71 ± 14.93 a,b,c	0.64 ± 0.08 a	Moderate	1504.58 ± 300.00 b,c
		Fraction 3	48.97 ± 9.09 a,b	1.02 ± 0.11 a,b	Strong	1188.85 ± 229.66 a,b,c
*P. angolensis*	Barks	Crude extract	7.11 ± 1.19 a	6.21 ± 0.33 d	Very strong	526.91 ± 101.74 a,b
		Fraction 1	165.84 ± 7.09 b,c	0.33 ± 0.08 a	Poor	4396.88 ± 226.40 e
		Fraction 2	74.39 ± 9.01 a,b,c	0.63 ± 0.05 a	Moderate	3021.61 ± 522.29 d
		Fraction 3	11.30 ± 1.90 a	4.46 ± 0.16 c	Very strong	2162.85 ± 44.19 c,d
	Leaves	Crude extract	8.67 ± 1.22 a	5.09 ± 0.32 c	Very strong	578.55 ± 18.85 a,b
		Fraction 1	21.33 ± 3.19 a	2.00 ± 0.43 b	Strong	1480.34 ± 252.16 a,b,c,f
		Fraction 2	10.46 ± 2.38 a	4.29 ± 0.02 c	Very strong	757.36 ± 68.61 a,b
		Fraction 3	10.45 ± 1.74 a	4.72 ± 0.11 c	Very strong	583.24 ± 130.35 a,b

^1^ Mean values in a column with different letters (a to f) are significantly different (*p*-value < 0.05); AAI—antioxidant activity index, DPPH—2,2-diphenyl-1-picrylhydrazyl.

**Table 7:** Anti-inflammatory and cytotoxicity results.

Plant Species	Plant Part	Samples	Anti-Inflammatory Activity—IC_50_ (mg/L) ^1^	Cytotoxicity		
				Concentration (mg/L)	Cell Viability (%) ^2^	*p*-Value ^3^
Negative Control ^a^					100 (100–100)	-
*J. paniculata*	Barks	Crude extract	784.24 ± 73.25 a	250 ^b^	74.90 (71.95–91.91)	0.037 ^a,b^ *
				500 ^c^	103.62 (70.34–112.75)	0.487 ^a,c^
				750 ^d^	74.09 (70.87–94.23)	0.037 ^ad^ *
		Fraction 3	274.86 ± 30.12 b	250 ^e^	76.24 (64.97–87.52)	0.053 ^a,e^
				500 ^f^	82.69 (68.99–91.54)	0.037 ^a,f^ *
				750 ^g^	77.05 (70.87–83.22)	0.053 ^a,g^
*P. angolensis*	Leaves	Crude extract	N.D.	250 ^h^	59.60 (50.20–64.70)	0.037 ^a,h^ *
				500 ^i^	54.50 (48.05–75.44)	0.037 ^a,i^ *
				750 ^j^	53.69 (51.01–63.90)	0.037 ^a,j^ *
		Fraction 3	N.D.	250 ^k^	63.89 (52.89–81.88)	0.037 ^a,k^ *
				500 ^l^	58.79 (56.11–66.56)	0.037 ^a,l^ *
				750 ^m^	91.01 (84.30–99.33)	0.037 ^a,m^ *

^1^ Mean values in a column with different letters (a and b) are significantly different (*p*-value < 0.05); ^2^ Results expressed as median and range; ^3^ Upper letters (a to m) were used to identify the statistical comparisons, * indicates significant results (*p*-value < 0.05); N.D.—not detected.

**Table 8:** Calculated mean difference between the distance of the injury of the negative control and the samples.

Plant Species	Plant Part	Samples	Concentration (mg/L)	Mean Difference (Negative Control—Sample)	95% C.I.	*p*-Value
*J. paniculata*	Barks	Crude extract	250	0.544	0.297–0.792	<0.001 *
			500	0.422	0.175–0.670	<0.001 *
			750	0.333	0.086–0.581	0.001 *
		Fraction 3	250	0.644	0.397–0.892	<0.001 *
			500	0.533	0.286–0.781	<0.001 *
			750	0.656	0.408–0.903	<0.001 *
*P. angolensis*	Leaves	Crude extract	250	0.344	0.097–0.592	0.001 *
			500	0.300	0.053–0.547	0.006 *
			750	0.389	0.142–0.636	<0.001 *
		Fraction 3	250	0.622	0.375–0.870	<0.001 *
			500	1.189	0.942–1.436	<0.001 *
			750	0.656	0.408–0.903	<0.001 *

C.I.—Confidence interval; * Indicates significant results (*p*-value < 0.05).

**Table 9:** Microscopic images obtained from the scratch wound-healing assay with the selected samples of *J. paniculata* (Magnification: 100×).

Representative Image of the Cells at the Initial Moment (0 h)	Samples	2 h	24 h
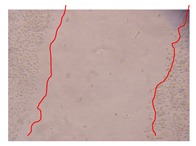	Negative control	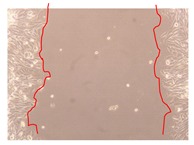	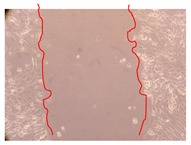
	*J. paniculata* barks crude extract (250 mg/L)	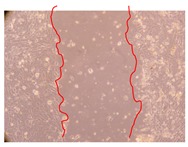	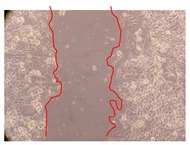
	*J. paniculata* barks crude extract (500 mg/L)	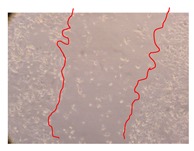	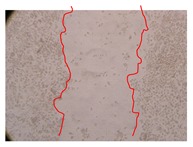
	*J. paniculata* barks crude extract (750 mg/L)	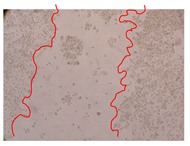	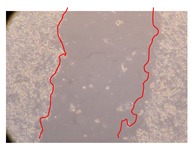
	*J. paniculata* barks Fraction 3 (250 mg/L)	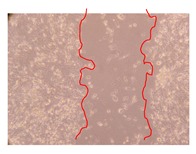	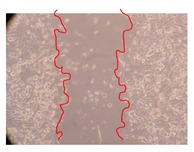
	*J. paniculata* barks Fraction 3 (500 mg/L)	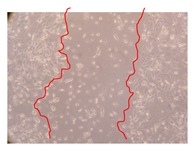	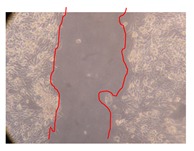
	*J. paniculata* barks Fraction 3 (750 mg/L)	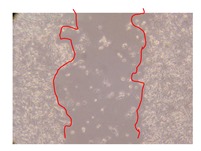	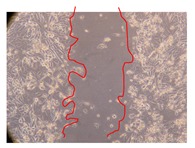

**Table 10:** Microscopic images obtained from the scratch wound-healing assay with the selected samples of *P. angolensis* (Magnification: 100×).

Samples	2 h	24 h
*P. angolensis* leaves crude extract (250 mg/L)	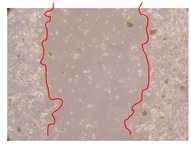	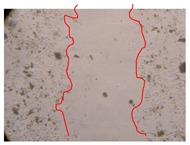
*P. angolensis* leaves crude extract (500 mg/L)	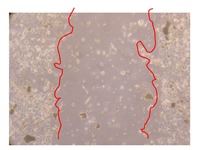	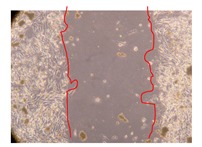
*P. angolensis* leaves crude extract (750 mg/L)	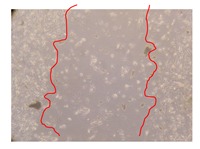	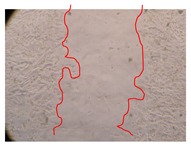
*P. angolensis* leaves Fraction 3 (250 mg/L)	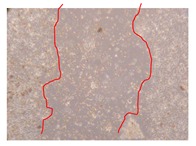	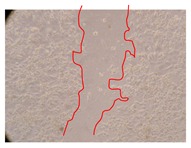
*P. angolensis* leaves Fraction 3 (500 mg/L)	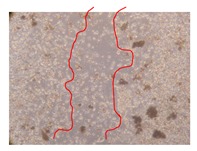	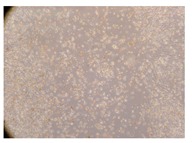
*P. angolensis* leaves Fraction 3 (750 mg/L)	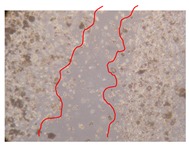	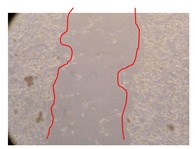

**Table 11:** Chromatographic conditions (retention time and wavelength) of the selected phenolic compounds.

Phenolic Compounds	Retention Time (min)	Wavelength (nm)
Gallic acid	3.826	280
Chlorogenic acid	10.710	322
Caffeic acid	13.540	322
Vanillic acid	13.597	263
Syringic acid	16.171	280
*p*-Coumaric acid	20.774	291
Taxifolin	24.973	291
Rutin	25.569	255
Ferulic acid	25.865	322
Ellagic acid	27.266	255
Rosmarinic acid	32.852	322
Quercetin	41.964	360

## Supplementary Materials

The following are available online, Figure S1: Use of medicinal plants according to age (years) (A), gender (B) and relation with the province (C); Figure S2: Use of medicinal plants according to forms of obtention (A) and season of collection (B); Figure S3: Use of medicinal plants according to source of information (A) and plant conservation mode (B); Figure S4: Most cited taxa (A) and therapeutic indications (B); Figure S5: Use of medicinal plants according to preparation method (A) and used part (B); Table S1: Informant consensus factor (ICF); Table S2: Fidelity level (FL) values for the most cited medicinal plants; Table S3: List of the medicinal plants cited and their relative frequency citation (RFC) and use value (UV).

## References

[1] Bibi T., Ahmad M., Tareen R.B., Tareen N.M., Jabeen R., Rehman S.U., Sultana S., Zafar M., Yaseen G. (2014). Ethnobotany of medicinal plants in district Mastung of Balochistan province-Pakistan. J. Ethnopharmacol..

[2] Novais M.H., Santos I., Mendes S., Pinto-Gomes C. (2004). Studies on pharmaceutical ethnobotany in Arrabida Natural Park (Portugal). J. Ethnopharmacol..

[3] Chipinga J.V., Kamanula J.F., Moyo P.B.B. (2018). Efficacy of pterocarpus angolensis crude extracts against Candida krusei, Staphylococcus aureus, Streptococcus agalactiae and Escherichia coli. Malawi Med. J..

[4] Bouasla A., Bouasla I. (2017). Ethnobotanical survey of medicinal plants in northeastern of Algeria. Phytomedicine.

[5] Camejo-Rodrigues J., Ascensão L., Bonet M.À., Vallès J. (2003). An ethnobotanical study of medicinal and aromatic plants in the Natural Park of ‘Serra de São Mamede’ (Portugal). J. Ethnopharmacol..

[6] Scartezzini P., Speroni E. (2000). Review on some plants of Indian traditional medicine with antioxidant activity. J. Ethnopharmacol..

[7] Mbosso E.J.T., Ngouela S., Nguedia J.C.A., Beng V.P., Rohmer M., Tsamo E. (2010). In vitro antimicrobial activity of extracts and compounds of some selected medicinal plants from Cameroon. J. Ethnopharmacol..

[8] Soares M.O., Vinha A.F., Coutinho F., Pires P.C. (2013). Antimicrobial natural products. Formatex.

[9] Chidumayo E.N. (2016). Biotic interactions, climate and disturbance underlie the distribution of two *Julbernardia* tree species in miombo woodlands of Africa. J. Trop. Ecol..

[10] Shewry P.R., Fowden L. (1976). 4,5-Dihydrpxypipecolic acids in the seed of *Julbernardia*, *Isoberlinia* and *Brachystegia*. Phytochemistry.

[11] Chungu D., Muimba-Kankolongo A., Roux J., Malambo F.M. (2007). Bark removal for medicinal use predisposes indigenous forest trees to wood degradation in Zambia. South. Hemisph. J..

[12] Abubakar M., Majinda R. (2016). GC-MS Analysis and Preliminary Antimicrobial Activity of *Albizia adianthifolia* (Schumach) and *Pterocarpus angolensis* (DC). Medicines.

[13] Afonso C.M.I., Gonçalves T.A.P., Muñiz G.I.B., Matos J.L.M., Nisgoski S. (2015). Mozambiques’s charcoals: Anatomy of nine native species. Bosque.

[14] Lumbile A.U., Kwerepe B.C., Kelathilwe M. (2007). The Characteristics and Economic Importance of *Pterocarpus angulensis* in D. C. Botswana. Pak. J. Biol. Sci..

[15] Chichinye A., Geldenhuys C.J., Chirwa P.W. (2019). Land-use impacts on the composition and diversity of the *Baikiaea*–*Guibourtia*–*Pterocarpus* woodlands of north-western Zimbabwe. South. For. J. For. Sci..

[16] Sadiki T.S., Tshisikhawe M.P. (2018). The ethnobotany of *Pterocarpus angolensis* DC.: A reflection of rural Venda speaking community of Gundani in Limpopo Province, South Africa. South Afr. J. Bot..

[17] Samie A., Housein A., Lall N., Meyer J.J.M. (2009). Crude extracts of, and purified compounds from, *Pterocarpus angolensis*, and the essential oil of *Lippia javanica*: Their in vitro cytotoxicities and activities against selected bacteria and *Entamoeba histolytica*. Ann. Trop. Med. Parasitol..

[18] Cai M., Lv H., Cao C., Zhang L., Cao R., Xu B. (2019). Evaluation of antimicrobial activity of *Pterocarpus* extracts. Ind. Crop. Prod..

[19] Zininga T., Anokwuru C., Sigidi M., Tshisikhawe M., Ramaite I., Traoré A., Heinrich H., Shondai A., Potgieter N. (2017). Extracts Obtained from *Pterocarpus angolensis* DC and *Ziziphus mucronata* Exhibit Antiplasmodial Activity and Inhibit Heat Shock Protein 70 (Hsp70) Function. Molecules.

[20] Kolodziejczyk-Czepas J. (2012). *Trifolium* species-derived substances and extracts—Biological activity and prospects for medicinal applications. J. Ethnopharmacol..

[21] Dhakad A.K., Pandey V.V., Beg S., Rawat J.M., Singh A. (2018). Biological, medicinal and toxicological significance of *Eucalyptus* leaf essential oil: A review. J. Sci. Food Agric..

[22] Elansary H.O., Salem M.Z.M., Ashmawy N.A., Yessoufou K., El-Settawy A.A.A. (2017). In vitro antibacterial, antifungal and antioxidant activities of *Eucalyptus* spp. leaf extracts related to phenolic composition. Nat. Prod. Res..

[23] Rababah T.M., Ereifej K.I., Esoh R.B., Al-u’datt M.H., Alrababah M.A., Yang W. (2011). Antioxidant activities, total phenolics and HPLC analyses of the phenolic compounds of extracts from common Mediterranean plants. Nat. Prod. Res..

[24] Cushnie T.P.T., Lamb A.J. (2005). Antimicrobial activity of flavonoids. Int. J. Antimicrob. Agents.

[25] Luís Â., Neiva D., Pereira H., Gominho J., Domingues F., Duarte A.P. (2014). Stumps of *Eucalyptus globulus* as a Source of Antioxidant and Antimicrobial Polyphenols. Molecules.

[26] U.S. Food and Drug Administration (2013). Guidance for Industry: Bioanalytical Method Validation. https://www.fda.gov/downloads/drugs/guidances/ucm368107.pdf.

[27] Andre C.M., Hausman J.-F., Guerriero G. (2016). *Cannabis sativa*: The Plant of the Thousand and One Molecules. Front. Plant Sci..

[28] Seely K., Lapoint J., Moran J., Fattore L. (2012). Spice drugs are more than harmless herbal blends: A review of the pharmacology and toxicology of synthetic cannabinoids. Prog. Neuropsychopharmacol. Biol. Psychiatry.

[29] Javanmardi J. (2003). Antioxidant activity and total phenolic content of Iranian *Ocimum accessions*. Food Chem..

[30] Mishra K., Ojha H., Chaudhury N.K. (2012). Estimation of antiradical properties of antioxidants using DPPH assay: A critical review and results. Food Chem..

[31] Dawidowicz A.L., Olszowy M. (2010). Influence of some experimental variables and matrix components in the determination of antioxidant properties by β-carotene bleaching assay: Experiments with BHT used as standard antioxidant. Eur. Food Res. Technol..

[32] García-Lafuente A., Moro C., Manchón N., Gonzalo-Ruiz A., Villares A., Guillamón E., Rostagno M., Mateo-Vivaracho L. (2014). In vitro anti-inflammatory activity of phenolic rich extracts from white and red common beans. Food Chem..

[33] Kar B., Kumar R.B.S., Karmakar I., Dola N., Bala A., Mazumder U.K., Hadar P.K. (2012). Antioxidant and in vitro anti-inflammatory activities of *Mimusops elengi* leaves. Asian Pac. J. Trop. Biomed..

[34] Naseri-Nosar M., Ziora Z.M. (2018). Wound dressings from naturally-occurring polymers: A review on homopolysaccharide-based composites. Carbohydr. Polym..

[35] Felician F.F., Yu R.H., Li M.Z., Li C.J., Chen H.Q., Jiang Y., Tiang T., Qi W.Y., Xu H.M. (2019). The wound healing potential of collagen peptides derived from the jellyfish *Rhopilema esculentum*. Chin. J. Traumatol. Engl. Ed..

[36] Talekar Y.P., Apte K.G., Paygude S.V., Tondare P.R., Parab P.B. (2017). Studies on wound healing potential of polyherbal formulation using in vitro and in vivo assays. J. Ayurveda Integr. Med..

[37] Alam P., Shakeel F., Anwer M.K., Foudah A.I., Alqarni M.H. (2018). Wound Healing Study of *Eucalyptus* Essential Oil Containing Nanoemulsion in Rat Model. J. Oleo Sci..

[38] Province Profile (2015). Governo de Angola. http://www.huila.gov.ao/InformacoesProvinciais.aspx?tipo=Perfil.

[39] Parekh J., Jadeja D., Chanda S. (2005). Efficacy of Aqueous and Methanol Extracts of Some Medicinal Plants for Potential Antibacterial Activity. Turk. J. Biol..

[40] Qi G., Yang L., Xiao C., Shi J., Mi Y., Liu X. (2015). Nutrient values and bioactivities of the extracts from three fern species in China: A comparative assessment. Food Funct..

[41] Pinho P.M., Naegchomnong W., Kijoa A., Nazareth N., Silva A.M.S., Eaton G., Herz W. (2006). An unusual glucoside from *Cleistanthus gracilis*. Phytochemistry.

[42] Silva O., Viegas S., Mello-Sampayo C., Costa M.J.P., Serrano R., Cabrita J., Gomes E.T. (2012). Anti-*Helicobacter pylori* activity of *Terminalia macroptera* root. Fitoterapia.

[43] Luís Â., Sousa S., Duarte A.P., Pereira L., Domingues F. (2018). Phytochemical characterization, and evaluation of rheological and antioxidant properties of commercially available juices of berries. J. Berry Res..

[44] Scherer R., Godoy H.T. (2009). Antioxidant activity index (AAI) by the 2,2-diphenyl-1-picrylhydrazyl method. Food Chem..

[45] Luís Â., Sousa S., Wackerlig J., Dobusch D., Duarte A.P., Pereira L., Domingues F. (2019). Star anise (*Illicium verum* Hook. f.) essential oil: Antioxidant properties and antibacterial activity against *Acinetobacter baumannii*. Flavour Fragr. J..

[46] Luís Â., Breitenfeld L., Ferreira S., Duarte A.P., Domingues F. (2014). Antimicrobial, antibiofilm and cytotoxic activities of *Hakea sericea* Schrader extracts. Pharm. Mag..

[47] Liang C.-C., Park A.Y., Guan J.-L. (2007). In vitro scratch assay: A convenient and inexpensive method for analysis of cell migration *in vitro*. Nat. Protoc..

