# Supplementary material for: Julbernardia paniculata and Pterocarpus angolensis: From Ethnobotanical Surveys to Phytochemical Characterization and Bioactivities Evaluation
Source: Molecules. 2020 Apr 16;25(8):1828. doi: 10.3390/molecules25081828 (PMC7221604; doi:10.3390/molecules25081828)
Supplement: Supplementary file 1 [file molecules-25-01828-s001.pdf]

Supplementary material

Julbernardia paniculata and *Pterocarpus angolensis*: from Ethnobotanical Surveys to Phytochemical Characterization and Bioactivities Evaluation

Eugénia Solange Santos [1,2,†], Ângelo Luís [1,3,†], Joana Gonçalves [1,3], Tiago Rosado [1,3,4], Luísa Pereira [5], Eugenia Gallardo [1,3] and Ana Paula Duarte [1,3,*]

[1] Centro de Investigação em Ciências da Saúde (CICS-UBI), Universidade da Beira Interior, Avenida Infante D. Henrique, 6200-506 Covilhã, Portugal; eugenia.santos@ubi.pt (E.S.S.); angelo.luis@ubi.pt (Â.L.); janitagoncalves@hotmail.com (J.G.); tiagorosadofful@hotmail.com (T.R.); egallardo@fcsaude.ubi.pt (E.G.)

[2] Instituto Superior Politécnico da Huíla, Universidade Mandume Ya Ndemufayo, Bairro Comercial, Avenida Hoji Ya Henda N.º 30, Caixa Postal N.º 201, Lubango, Huíla, República de Angola

[3] Laboratório de Fármaco-Toxicologia, UBIMedical, Universidade da Beira Interior, Estrada Municipal 506, 6200-284 Covilhã, Portugal

[4] C4 - Cloud Computing Competence Centre, UBIMedical, Universidade da Beira Interior, Estrada Municipal 506, 6200-284 Covilhã, Portugal

[5] Centro de Matemática e Aplicações (CMA-UBI), Universidade da Beira Interior, Rua Marquês d'Ávila e Bolama, 6201-001 Covilhã, Portugal; lpereira@ubi.pt

* Correspondence: apcd@ubi.pt; Tel.: +351-275-329-099

† These authors contributed equally to this work.

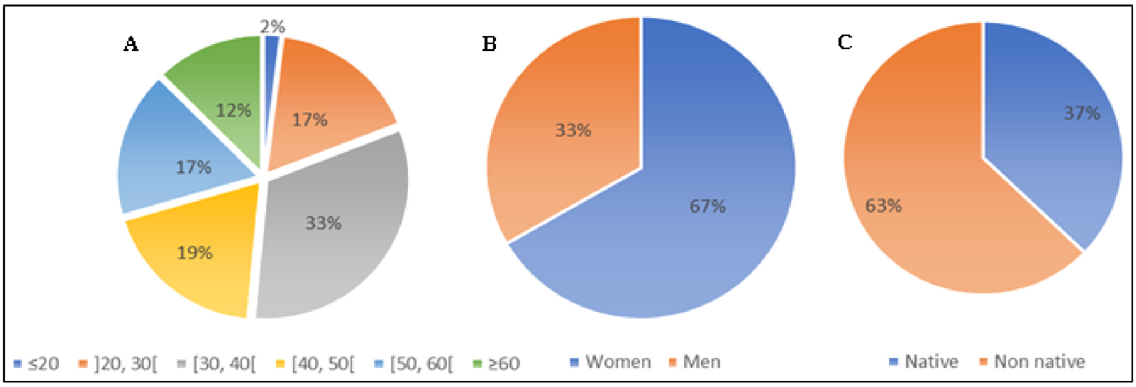

Figure S1. Use of medicinal plants according to age (years) (A), gender (B) and relation with the province (C).

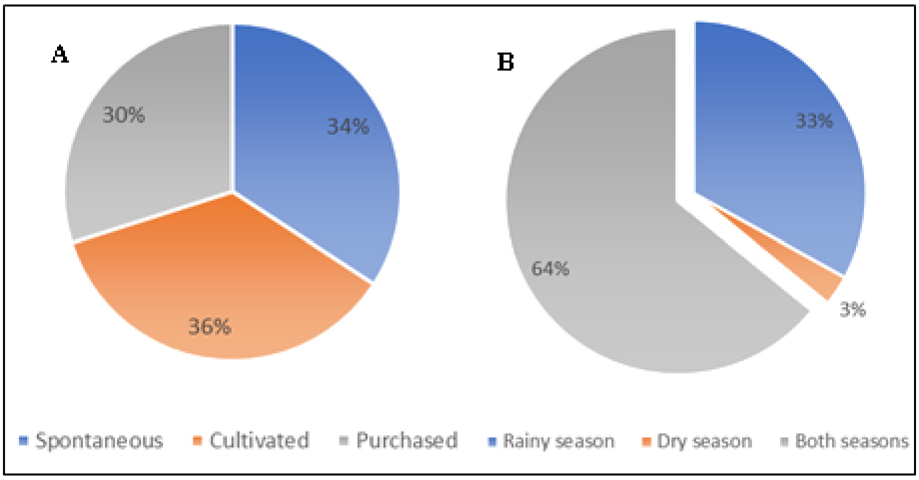

Figure S2. Use of medicinal plants according to forms of obtention (A) and season of collection (B).

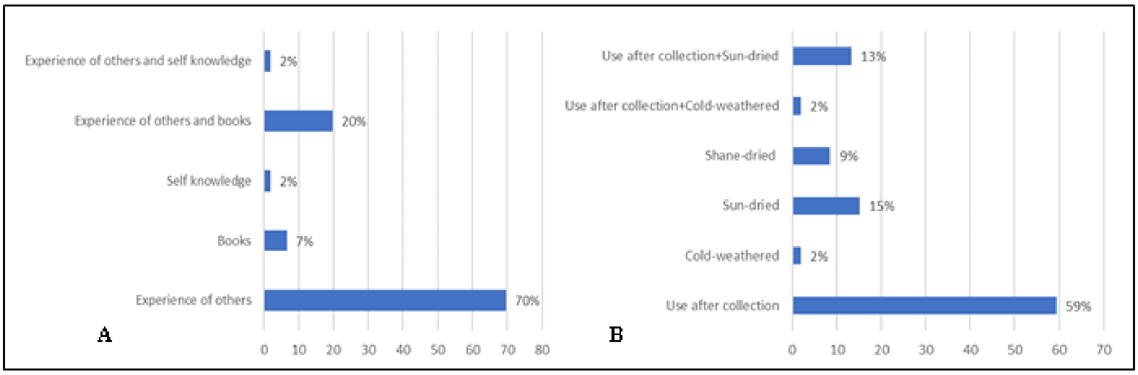

Figure S3. Use of medicinal plants according to source of information (A) and plant conservation mode (B).

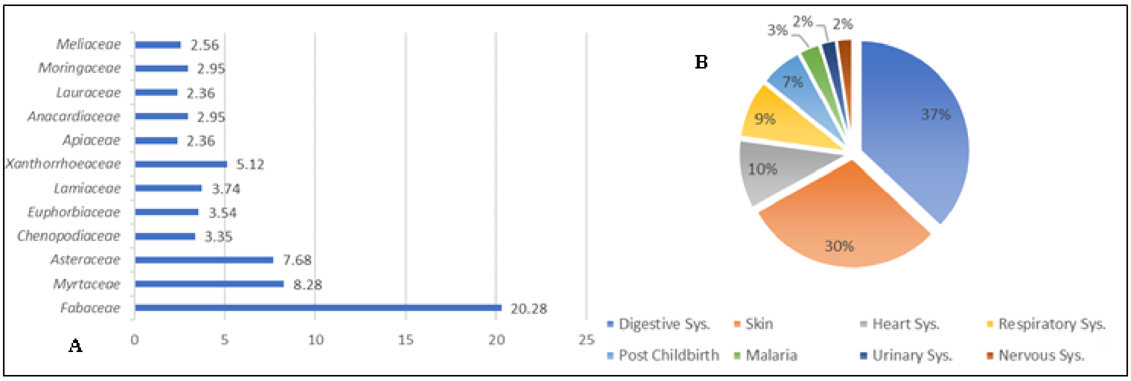

Figure S4. Most cited taxa (A) and therapeutic indications (B).

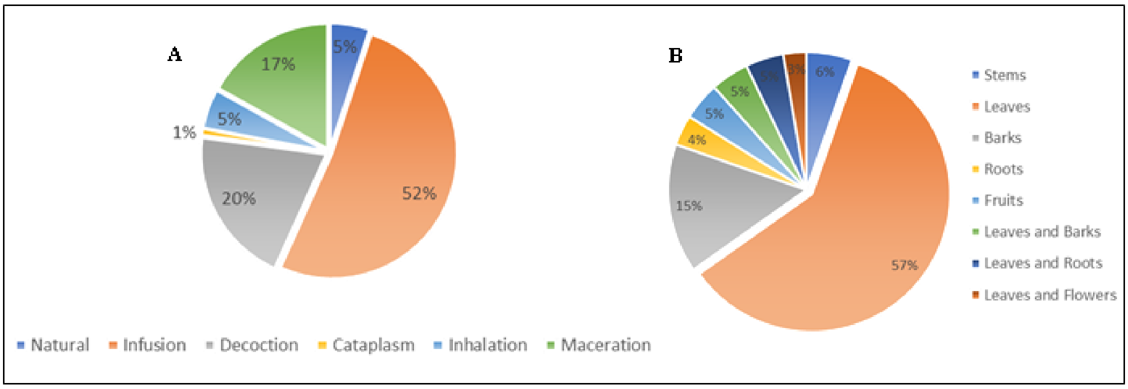

Figure S5. Use of medicinal plants according to preparation method (A) and used part (B).

Statistical quantitative analysis of the ethnobotanical results

The informant consensus factor (ICF) values of the medicinal plants reported in the ethnobotanical surveys ranged from 0 to 1. The recorded traditional uses were grouped by therapeutic indications (Table S1).

Table S1. Informant consensus factor (ICF).

Therapeutic indications	Number of used reports	Number of taxa	ICF
Digestive system	74	18	0.63
Skin disorders	31	10	0.70
Respiratory system	12	6	0.55
Cardiac system	13	3	0.83
Urinary system	3	3	0.00
Nervous system	3	3	0.00
Post childbirth complications	8	3	0.71

The highest ICF (0.83) was verified for cardiac system, followed by post childbirth complications (0.71), skin disorders (0.70), digestive system (0.63) and respiratory system (0.55). These results may be explained by the ability of the interviewed people in diagnosing those categories of pathologies. Interestingly, ICF equal to 0.00 was obtained for both urinary and nervous systems, indicating that the informants disagree in which plants should use or even lack of exchange of information about the use of the plants.

The preference of the Huíla province inhabitants to use some specific medicinal plants due to their therapeutic indications was established by high fidelity level (FL) values (Table S2). *J. paniculata* and *Eucalyptus* spp. presented the highest FL (100.0%), being used to treat skin disorders and respiratory system problems, respectively. *J. paniculata* is ethnobotanically used to repair skin lesions and to regenerate damaged tissues; infusions of the barks of this plant species are frequently employed. *Eucalyptus* species are often used in folk medicine for respiration stimulation, relieve coughing, and help to expel mucus and relax the respiratory muscles [1,2]. Leaf extracts of *Eucalyptus* species have been traditionally used to treat colds, asthma and coughs [2].

P. angolensis also showed a high FL value (93.3%), being mainly used for digestive system ailments. Additionally, this plant species is often used for cardiac system diseases (FL=76.9%), and for skin disorders (FL=20.0%). This plant is mentioned to be used in traditional medicine to treat gastro-intestinal problems [3,4], which is confirmed by the FL values now obtained.

Table S2. Fidelity level (FL) values for the most cited medicinal plants.

Therapeutic indications	Plant species	FL (%)
Digestive system	*Aloe vera*	57.1
	Artemisia vulgaris	80.0
	Moringa ovalifolia	60.0
	Pterocarpus angolensis	93.3
	Psidium guajava	80.0
Skin disorders	*Aloe vera*	42.9
	Artemisia vulgaris	30.0
	Julbernardia paniculata	100.0
	Moringa ovalifolia	40.0
	Pterocarpus angolensis	20.0
	Psidium guajava	26.7
Respiratory system	*Cupressus lusitanica*	75.0
	Eucalyptus spp.	100.0
Cardiac system	*Pterocarpus angolensis*	76.9

Relative frequency citation (RFC) values were determined for the most cited plant species (Table S3). The highest values were obtained for *J. paniculata* (0.12264) and *P. angolensis* (0.14151), which indicates that these two species are popular amongst the respondents and suggest their medicinal potential, encouraging also further research to clarify their chemical composition and biological activities.

The use value (UV) was also calculated, and the values are summarized in Table S3. The plants that presented the highest UV were once again *J. paniculata* (1.85) and *P. angolensis* (2.13), meaning that these plants are widely used in the treatment of several diseases.

From the experience obtained in the field interviews it is possible to note that *J. paniculata* and *P. angolensis* are often mentioned by the users that collect them not only to their own utilization but also to sell it in local markets.

Table S3. List of the medicinal plants cited and their relative frequency citation (RFC) and use value (UV).

Plant species	Number of participants that cited the species	RFC	UV
Aloe vera	7	0.06604	1.45
Anacardium occidentale	1	0.00943	1
Arnica chamissonis	1	0.00943	1
Artemisia afra	1	0.00943	1
Artimisia vulgaris	10	0.09434	1.1
Azadirachta indica	2	0.01887	1.5
Bauhinia forficata	1	0.00943	1
Carica papaya	1	0.00943	1
Cochlospermum angolensis	2	0.01887	1.5
Cupressus lusitanica	4	0.03774	1
Curcubita pepo	1	0.00943	2
Dysphania ambrosioides	2	0.01887	1
Englerophytum magalismontanum	1	0.00943	1
Erigeron canadensis	1	0.00943	1
Eucalyptus spp.	4	0.03774	1
Eugenia uniflora	1	0.00943	1
Indigofera spp.	2	0.01887	1
Julbernardia paniculata	13	0.12264	1.85
Licopersicon esculentum	1	0.00943	1
Lippia javanica	1	0.00943	1
Mangifera indica	3	0.02830	1
Mentha × rotundifolia	2	0.01887	1
Moringa ovalifolia	5	0.04717	1.6
Nasturtium officinale	1	0.00943	1
Ocimum gratissimum	3	0.02830	1
Olea europaea L. subsp. *africana*	2	0.01887	1
Opuntia ficus-indica	1	0.00943	1
Parinari curatellifolia	1	0.00943	1
Passiflora edulis	1	0.00943	1
Pseudeminia benguellensis	1	0.00943	1
Psidium guajava	5	0.04717	1.2
Pterocarpus angolensis	15	0.14151	2.13
Punica granatum	3	0.02830	1
Ricinus communis	1	0.00943	1
Rosmarinus officinalis	1	0.00943	1
Sambucus nigra	1	0.00943	1
Sopubia spp.	1	0.00943	2
Vernonia amygdalina	1	0.00943	1
Zea mays	1	0.00943	1

References

1. Dhakad, A.K.; Pandey, V.V.; Beg, S.; Rawat, J.M.; Singh, A. Biological, medicinal and toxicological significance of *Eucalyptus* leaf essential oil: a review. *J. Sci. Food Agric.* **2018**, *98*, 833–848.

2. Elansary, H.O.; Salem, M.Z.M.; Ashmawy, N.A.; Yessoufou, K.; El-Settawy, A.A.A. *In vitro* antibacterial, antifungal and antioxidant activities of *Eucalyptus* spp. leaf extracts related to phenolic composition. *Nat. Prod. Res.* **2017**, *31*, 2927–2930.

3. Abubakar, M.; Majinda, R. GC-MS Analysis and Preliminary Antimicrobial Activity of *Albizia adianthifolia* (Schumach) and *Pterocarpus angolensis* (DC). *Medicines*, *3*, 3, doi:10.3390/medicines3010003.

4. Samie, A.; Housein, A.; Lall, N.; Meyer, J. J. M. Crude extracts of, and purified compounds from, *Pterocarpus angolensis*, and the essential oil of *Lippia javanica*: Their *in vitro* cytotoxicities and activities against selected bacteria and *Entamoeba histolytica*. *Ann. Trop. Med. Parasitol.* **2009**, *103*, 427–439.
